# Genome wide association study of passive immunity and disease traits in beef-suckler and dairy calves on Irish farms

**DOI:** 10.1038/s41598-020-75870-4

**Published:** 2020-11-04

**Authors:** Dayle Johnston, Robert Mukiibi, Sinéad M. Waters, Mark McGee, Carla Surlis, Jennifer C. McClure, Matthew C. McClure, Cynthia G. Todd, Bernadette Earley

**Affiliations:** 1 Animal and Grassland Research and Innovation Centre, Teagasc, Grange, Dunsany, C15 PW93 Co. Meath Ireland; 2 The Roslin Institute and Royal (Dick) School of Veterinary Studies, University of Edinburgh, Edinburgh, EH25 9RG UK; 3 Irish Cattle Breeding Federation, Cork, P72 X050 Ireland

**Keywords:** Genetics, Zoology, Biomarkers, Diseases

## Abstract

Calves with lower concentrations of immunoglobulin G (IgG) in their blood, have a greater risk of developing diseases. There is a lack of knowledge on genetic markers known to be associated with immunological variability or disease resistance. Therefore, the objective of this study was to identify SNP markers associated with passive immunity measures (serum IgG, serum protein, albumin, globulin and total protein concentrations, total solids Brix percentage, zinc sulphate turbidity units) and disease (pneumonia, diarrhoea, crude illness) traits in Irish commercial beef-suckler and dairy calves through genome wide association studies (GWAS). Genotyping was performed on DNA samples from beef-suckler (n = 698) and dairy (n = 1178) calves, using the IDBv3 chip. Heritability of passive immunity associated traits (range 0.02–0.22) and the disease traits (range 0.03–0.20) were low-to-moderate. Twenty-five and fifteen SNPs approached genome wide significance (*P* < 5 × 10^−5^) for the passive immunity and the disease traits, respectively. One SNP “ARS-BFGL-BAC-27914” reached Bonferroni genome wide significance (*P* < 1.15 × 10^−6^) for an association with serum IgG concentration in beef calves. Further work will evaluate these SNPs in larger cattle populations and assess their contribution to genomic selection breeding strategies, aimed towards producing more disease resistant livestock.

## Introduction

High morbidity and mortality rates in beef and dairy calves result in significant economic losses for farmers and a reduction in animal welfare^1^. Internationally, calf mortality rates in the first year of life range from 5 to 11% in dairy and beef enterprises^2–4^. In Ireland, the combined mortality rate of beef and dairy calves from 0 to 12 months of age is 5.8%^5^. Furthermore, in a large scale, Irish observational study, Todd et al.^6^ reported that 20% of beef-suckler calves and 30% of dairy calves were treated for at least one disease event, from birth to 6 months of age, and higher treatment rates have been observed internationally^7^. Internationally, the diseases responsible for the majority of the morbidity and mortality, in beef and dairy calves less than 6 months of age, are diarrhoea and pneumonia^4,8–10^. Calves with lower plasma or serum immunoglobulin G (IgG) concentrations, or failure of passive transfer are at a greater risk of developing these diseases^6,11–13^. Since there is no trans-placental transfer of immunoglobulins or leukocytes in cattle, the calf is born without detectable antibodies^13–15^. Immunoglobulins and other macromolecules (e.g. maternal leukocytes, growth factors, hormones, cytokines) in colostrum ingested by the calf after birth, are absorbed and transported through enterocytes, and subsequently deposited into the circulatory system of the neonatal calf in the first 24 h of life^14^. There are many different tests that can assess passive transfer, some of which measure IgG directly (IgG ELISA) and others that act as a proxy for IgG concentration, which include serum protein, albumin, globulin and total protein concentrations, total solids Brix percentage, and zinc sulphate turbidity (ZST) units^6^. Passive immunity test results are generally categorised for failure of passive transfer (FPT) using test-specific cut-off values^13^.

The efficiency of passive transfer is strongly dependent on genetic, environmental and management factors such as the quantity and the quality of colostrum that the calf receives, and the length of time from birth to colostrum ingestion^13,16,17^. Differences between breeds in serum immunoglobulin concentration of neonatal calves have been observed^18–22^. We have previously observed that genes involved in the blood systemic immune response, particularly in the development of immune competence, are differentially expressed between beef and dairy calves during the first week of life^22^. Additionally, susceptibility to pneumonia has previously been demonstrated to be heritable in pre-weaned Holstein calves in the United States^23,24^. A further point which illustrates a genetic basis for immunity in cattle is that natural antibody (IgG and IgM) concentration in serum of Canadian Holstein cows has been estimated to be moderately heritable and associated with several DNA marker variants^25^.

Breeding programs to improve the genetic merit of both production and health traits of livestock have been extremely successful, both internationally and in Ireland, particularly in the dairy sector^26–28^. An important goal of these programmes is to breed robust animals which display resistance to disease^29^. In Ireland, health traits (mastitis, somatic cell count and lameness) currently make up 4% of the Economic Breeding Index (EBI), which is the index used for the selection of profitable dairy cattle in Ireland^30^. The beef Euro-Star Index, a relatively new index incorporating two overall indexes, the Replacement Index and the Terminal Index, is used for the selection of profitable beef animals in Ireland. Currently, cow survival and calf mortality make up 8% and 1%, respectively, of the Replacement Index, and mortality makes up 3% of the Terminal Index^31^; however, disease resistance or improved immune function traits are not included. This is partly due to limited phenotypic reporting and the current lack of knowledge on genetic markers known to be associated with immunological variability or disease resistance, to incorporate into these genetic breeding programmes (e.g. EBI, beef Euro-Star Index). Consequently, the discovery and addition of SNPs conferring resistance to disease and to general improved immune response capabilities, including calf passive immunity, would be extremely beneficial to these Irish breeding indexes, as it may result in the augmentation of selection of healthier cattle. Therefore, the objectives of the current study were to perform GWAS for passive immunity and disease-related traits in Irish beef-suckler and artificially-reared dairy-bred calves, and to identify SNP markers associated with superior immunity and disease resistance.

## Methods

### Ethical approval

Project and individual authorisations, in accordance with European Union (Protection of Animals used for Scientific Purposes) Regulations 2012 (S.I. No. 543 of 2012) as amended and Directive 2010/63/EU, were obtained (Health Products Regulatory Authority, Dublin, Ireland (AE19132-P006)). All study procedures were also reviewed and approved by the Teagasc Animal Ethics Committee (TAEC-97).

### Animal details

Beef-suckler (n = 698) and dairy (n = 1178) calves used in this study were from commercial suckler beef (n = 29) and dairy farms (n = 32) in Ireland and were part of a larger study, examining passive immunity status in Irish suckler beef and dairy calves^6^. The suckler-bred calves included a mix of pure-bred and cross-bred Limousin, Simmental, Charolais, Aberdeen Angus, Hereford, Belgium Blue, Parthenaise, Saler, Shorthorn and Blonde d'Aquitaine. The dairy-bred calves included a mix of pure-bred and cross-bred Holstein–Friesian and Jersey, and beef × dairy breeds. All calves in the present study were born and resided at a beef or dairy farm in which at least ten calves per farm were available for genotyping.

### Passive immunity traits measurement and profiling

Passive immunity traits were profiled from analyses performed on the calves’ serum samples (total IgG, total protein, albumin, specific gravity, globulin, total solids percentage from a Brix refractometer, ZST). All serum sample analyses performed in this study have been described in detail^6^; however, for clarity, they are summarised briefly here. Blood samples were collected from heifer and bull calves, aged between 1 and 21 days (as the half-life for IgG in colostrum fed calves is 28.5 days^32^), by jugular venepuncture, into 8.5 ml vacutainers (BD Vacutainer Serum Separator Tube II Advance 367,958 no anticoagulant, Unitech, Dublin, Ireland) using an 18-gauge needle. Samples were allowed to clot and then stored at 4 °C for 24 h. Serum was harvested following centrifugation (1600 × g for 10 min at 4 oC) and subsequently frozen at− 20 °C.

Total IgG concentration was directly measured in the serum samples using a commercial ELISA (BIO K165 test kit, BioX Diagnostics, Jemelle, Belgium), as described by Dunn, et al*.*^33^. A clinical chemistry analyser (Olympus AU400, Tokyo, Japan) and test reagent kits (OSR6132 and OSR6102, Beckman Coulter Ireland Inc., Lismeehan, Co. Clare, Ireland) were used to quantitatively determine serum total protein and albumin concentrations, as described by Early, et al*.*^34^. Globulin concentration was calculated for each serum sample as the difference between the total protein and albumin concentration. Serum samples were analysed for ZST units, as described by McEwan et al*.*^35^. An optical Brix refractometer with automatic temperature compensation (RSG-100ATC, Grand Index Solution Enterprise Limited, Hong Kong, China) was used to determine total solids percentage by Brix refractometry. A digital hand held refractometer with automatic temperature compensation (DR-303, Index Instruments Ltd, Cambridgeshire, UK) was used to determine total protein concentration which was subsequently referred to as specific gravity.

### Health phenotypes for disease traits

Cases of calf pneumonia, diarrhoea, and any other illnesses during the first 6 months of life were observed and recorded by the farmers using standardised recording sheets^6^. Any calves which were sold before they reached 6 months of age or for which no data were received, were removed from the GWAS analyses. Health traits analysed by GWAS were crude illness, pneumonia and diarrhoea. Crude illness was defined as calves treated for at least one disease event, excluding injury, attributed to any cause (e.g. bovine respiratory disease (BRD), diarrhoea, navel infection, joint infection, lameness). Pneumonia was defined as calves treated for BRD and diarrhoea was defined as calves treated for diarrhoea.

### Animal genotyping

DNA was extracted from blood samples collected in 6 ml K_3_EDTA tubes (Vacuette; Cruinn Diagnostics, Ireland) using the Maxwell 16 Blood DNA kit (Promega, Madison, WI, USA) as per manufacturer’s instructions. Extracted DNA samples were analysed for quality and quantity using a Nanodrop spectrophotometer and normalised to 50 ng/µL for genotyping analysis. Genotyping was performed at Weatherby’s Scientific Ltd. (Johnstown, Naas, Co. Kildare, Ireland) using the IDB*v*3 chip which contains 50,855 markers^36^.

### Genotype quality control and population substructure correction

All traits were analysed within three separate analysis groups; combined analysis including all beef and dairy calves, dairy calves only analysis group and beef calves only analysis group. Quality control (QC) was carried out on genotypes within the three separate analysis groups using PLINK v1.90b3.44 64-bit^37^. SNPs were removed from the analyses if they had a genotype call rate of less than 0.95, a minor allele frequency of less than 0.05 or showed a significant (*P* value < 1 × e^−4^) deviation from Hardy–Weinberg equilibrium. In the beef and dairy calf combined analysis, no variants were removed due to poor genotype call rates, 2,084 variants were removed as they were out of Hardy–Weinberg equilibrium and 5,825 variants were removed due to minor allele thresholds < 0.05. Following QC in PLINK, 42,946 autosomal variants and 1,876 calves passed all filters and remained for further analysis. In the beef calf analysis, no variants were removed due to missing genotype data, 413 variants were removed as they were out of Hardy–Weinberg equilibrium and 6,854 variants were removed due to minor allele thresholds < 0.05. Following QC in PLINK, 43,588 autosomal variants and 698 calves passed all filters and remained for further analysis. In the dairy calf analysis, no variants were removed due to missing genotype data, 349 variants were removed as they were out of Hardy–Weinberg equilibrium and 6,291 variants were removed due to minor allele thresholds < 0.05. A further two calves were removed from the analysis as they had a recorded breed inconsistent with that of a dairy-bred animal. Following QC in PLINK, 44,215 autosomal variants and 1,176 calves passed all filters and remained for further analysis. The retained genotypes within each of the three analysis groups (combined beef and dairy calves, beef calves only and dairy calves only), were separated into four principal components based on breed population structure using PLINK v1.90b3.44 64-bit^37^. The principle components were used for population substructure correction of the phenotypic data.

### Correcting the phenotype traits for fixed and random effects

The quantitative phenotype distributions were initially visualized using histogram plots generated in Microsoft Excel in order to identify potential outlier records^38^. The phenotypic records which were more than three standard deviations away from the mean were excluded as outliers (Supplementary Table S1). Calves which were sold before they reached 6 months of age or for which no recording sheets were received were removed from all disease trait analyses (Supplementary Table S1).

Phenotype data were examined for significant fixed effects of population structure principal components, sex, age at blood sample collection, season of birth and task (herd level or calf level study as described by Todd, et al*.*^6^) using either a lmer model with the package lme4 version 1.1-18-1^39^ or a glmer model with the package mlmRev version 1.0-6^40^, in R version 3.5.1, for continuous and binary phenotypes, respectively. Non-significant fixed effects were sequentially removed from each phenotype model and the optimal model was selected for each phenotype by examining AIC values and R squared values (package MuMIn version 1.42.1)^41^ of the models (Supplementary Table S1). Phenotypes were corrected for significant fixed effects and the random effect of farm by obtaining the residuals of the optimal model for each phenotype and carrying these values forward for the GWAS.

### Genome-wide association studies and heritability analyses

Heritability estimates and GWAS analyses for each phenotype were performed using GCTA (version 1.91.6 beta1)^42^. The GWAS were carried out using the mixed linear model association (–mlma) method:

$$ {\text{y}}_{{{\text{ij}}}} = {\text{b}}_{{\text{j}}} {\text{SNP}}_{{{\text{ij}}}} + {\text{ gi }} + {\text{ e}}_{{{\text{ij}}}} ,{\text{ e}}\, \, \sim \,{\text{ N}}\left( {0,{\text{ I}}\sigma_{{\text{e}}}^{{2}} } \right) $$

where y_ij_ was the adjusted phenotype of the ith individual, b_j_ was the allele substitution effect of the jth SNP marker, SNP_ij_ was the genotype of the ith animal for the jth SNP (coded as 0, 1 and 2), g_i_ was the random polygenic effect of the ith individual, and e_ij_ was the random residual effect for the ith individual and jth SNP. The polygenic effects (g) followed a normal distribution g ~ N(0, Gσ_g_^2^), where G was the genomic relationship matrix (calculated as described by ^42^), and the residuals followed a normal distribution e ~ N(0, Iσ_e_^2^).

The GWAS resulted in the generation of association statistics for each trait of interest (total IgG, total protein, albumin, specific gravity, globulin, total solids percentage from a Brix refractometer, ZST units, crude illness, pneumonia, diarrhoea) within each analysis group (combined beef and dairy calves, beef calves only and dairy calves only). SNPs were considered significant at the genome wide threshold if they had a Bonferroni *P* value less than 0.05 (i.e. *P* value threshold = 0.05/total no. of variants in analysis), whereas SNPs with raw *P* value s < 5 × 10^−5^ were considered to be suggestively significant. Manhattan plots were generated in R (version 3.5.1) using the package qqman version 0.1.4^43^. Genes closest to SNPs of interest were obtained using the package Bedtools (version 2.27.1) closest^44^.

## Results

Mean, standard deviation, minimum and maximum values for the passive immunity traits in the combined beef-suckler and dairy calf population are shown in Table 1. In the combined analysis of beef-suckler and dairy calves, heritability estimates of the passive immunity associated traits and the disease traits were low-to-moderate (range 0.06–0.19) (Table 2). There were no SNPs which reached Bonferroni genome wide significance. However, there was one SNP in the serum IgG analysis, two SNPs in the albumin analysis, three SNPs in the total protein analysis, three SNPs in the globulin analysis, one SNP in the specific gravity analysis, five SNPs in the total solids percentage from a Brix refractometer analysis, four SNPs in the pneumonia analysis, one SNP in the diarrhoea analysis and two SNPs in the crude illness analysis, which were suggestively significant (*P* < 5 × 10^−5^) (Table 3).

**Table 1:** Means and standard deviations for the passive immunity traits in the Irish commercial beef-suckler and dairy calves.

Variable	Mean	S.D	Maximum	Minimum
Immunoglobulin G (mg/ml)	13.35	5.17	29.72	1.50
Albumin	27.08	2.56	34.80	18.30
Total protein (g/l)	61.76	8.10	86.20	38.40
Globulin (g/l)	34.58	8.65	61.20	12.40
Zinc sulphate turbidity (units)	16.32	5.80	34.10	0.30
Specific gravity (g/dl)	6.13	0.87	8.70	3.20
Total solids Brix (%)	8.94	0.93	11.60	6.00

*S.D* standard deviation.

**Table 2:** Heritability estimates for passive immunity and disease traits in Irish commercial beef-suckler and dairy calves.

Variable	Combined beef and dairy heritability	Combined beef and dairy S.E	Beef calves heritability	Beef calves S.E	Dairy calves heritability	Dairy calves S.E
Immunoglobulin G	0.16	0.05	0.1	0.09	0.15	0.06
Albumin	0.19	0.05	0.00	0.06	0.22	0.06
Total protein	0.12	0.04	0.05	0.07	0.13	0.06
Globulin	0.18	0.05	0.04	0.07	0.19	0.06
Zinc sulphate turbidity	0.05	0.04	0.03	0.07	0.00	0.03
Specific gravity	0.07	0.04	0.02	0.06	0.05	0.04
Total solids Brix %	0.06	0.04	0.00	0.05	0.05	0.05
Pneumonia	0.1	0.05	0.00	0.05	0.09	0.07
Diarrhoea	0.13	0.05	0.00	0.05	0.20	0.08
Crude illness	0.13	0.05	0.03	0.06	0.19	0.08

*S.E* standard error.

**Table 3:** Irish commercial beef-suckler and dairy calves GWAS results for passive immunity and disease traits.

Population	Variable	Associated SNP	Chromosome	RS number	*P* value	Closest gene	Distance of SNP to gene
Combined dairy and beef	Immunoglobulin G	ARS-BFGL-NGS-114208	1	rs110082431	3.76E−05*	*ENSBTAG00000045984*	25,324
	Albumin	ARS-BFGL-NGS-11531	12	rs109028090	7.87E−06	*DHRS12*	0
		ARS-BFGL-NGS-100170	12	rs109708871	2.68E−05	*SLC10A2*	− 462,315
	Total protein	BTB-01120104	4	rs42277262	1.75E−05	*GNAI1*	−36,497
		UA-IFASA-8558	24	rs41646027	3.92E−05	*LPIN2*	0
		BTB-00174357	4	rs43383611	4.22E−05	*KIAA1324L*	22,817
	Globulin	UA-IFASA-8558	24	rs41646027	3.17E−05	*LPIN2*	0
		BTB-01120104	4	rs42277262	3.34E−05	*GNAI1*	− 36,497
		BOVINEHD2400010261	24	rs109172808	4.44E−05	*LPIN2*	0
	Specific gravity	BTB-01120104	4	rs42277262	3.03E−06	*GNAI1*	− 36,497
	Total solids Brix %	ARS-BFGL-NGS-69831	7	rs42619441	2.71E−05	*ENSBTAG00000038284*	0
		BTB-01120104	4	rs42277262	3.89E−05	*GNAI1*	−36,497
		ARS-BFGL-NGS-15820	11	rs110788172	3.93E−05	*CDKL4*	0
		UA-IFASA-8558	24	rs41646027	4.05E−05	*LPIN2*	0
		BOVINEHD2400010261	24	rs109172808	4.80E−05	*LPIN2*	0
	Pneumonia	ARS-BFGL-NGS-57317	25	rs110476838	8.60E−06	*ENSBTAG00000014417*	334,478
		ARS-BFGL-NGS-50482	2	rs110785912	2.73E−05	*CXCR4*	447,880
		BOVINEHD2900007001	29	rs42465360	3.60E−05	*SLC6A5*	338,375
		HAPMAP52014-BTA-90653	5	rs41593661	3.75E−05	*ENSBTAG00000046268*	30,344
	Diarrhoea	ARS-BFGL-NGS-114897	11	rs110764285	4.40E−05	*NFU1*	1970
	Crude illness	ARS-BFGL-NGS-110312	12	rs110793235	3.77E−05	*KL*	− 74,444
		HAPMAP40647-BTA-110965	4	rs41575187	3.91E−05	*DYNC1I1*	0
Beef calves	Immunoglobulin G	ARS-BFGL-BAC-27914	20	rs110897405	2.20E−07	*PARP8*	0
		HAPMAP51687-BTA-114691	20	rs41616927	1.57E−05	*ISL1*	85,644
		ARS-BFGL-NGS-67929	2	rs110780508	3.56E−05	*MREG*	0
	Total protein	ARS-BFGL-BAC-27914	20	rs110897405	2.91E−05	*PARP8*	0
	Globulin	HAPMAP47694-BTA-67030	3	rs43710738	1.60E−05	*PTGFRN*	11,015
		ARS-BFGL-BAC-27914	20	rs110897405	2.99E−05	*PARP8*	0
		BTB-00212876	4	rs43420430	4.07E−05	*DPP6*	0
		BTA-03263-RS29011028	21	rs29011028	4.29E−05	*PPP2R5C*	20,692
	Zinc sulphate turbidity	BTA-47238-NO-RS	1	rs110704582	1.00E−05	*PLCH1*	− 144,011
		BTB-00212876	4	rs43420430	2.72E−05	*DPP6*	0
		HAPMAP47742-BTA-80071	7	rs41656596	4.51E−05	*RASA1*	0
		ARS-BFGL-NGS-55396	10	rs110351463	4.79E−05	*SYNE2*	0
	Specific gravity	ARS-BFGL-NGS-4066	26	rs109923400	4.53E−05	*ENSBTAG00000003529*	− 5054
	Crude illness	ARS-BFGL-NGS-114450	24	rs109440690	1.82E−05	*ATP8B1*	− 131,562
		BOVINEHD0900029149	9	rs109299906	2.62E−05	*QKI*	− 73,861
		ARS-BFGL-NGS-43453	8	rs110620477	4.58E−05	*SMARCA2*	0
Dairy calves	Immunoglobulin G	HAPMAP54718-RS29022960	9	rs29022960	1.56E−05	*ZNF292*	0
		UA-IFASA-8558	24	rs41646027	3.78E−05	*LPIN2*	0
		BOVINEHD2400010261	24	rs109172808	4.15E−05	*LPIN2*	0
	Albumin	ARS-BFGL-NGS-11531	12	rs109028090	1.48E−05	*DHRS12*	0
		ARS-BFGL-NGS-6195	18	rs109046420	2.27E−05	*BANP*	0
		HAPMAP39432-BTA-76145	6	rs41596019	2.74E−05	*STIM2*	− 184,275
	Total protein	UA-IFASA-8558	24	rs41646027	7.47E−06	*LPIN2*	0
		ARS-BFGL-NGS-83128	11	rs110743782	8.54E−06	*OTOF*	0
		BOVINEHD2400010261	24	rs109172808	9.62E−06	*LPIN2*	0
		ARS-BFGL-NGS-11057	11	rs109425927	2.17E−05	*OTOF*	0
	Globulin	UA-IFASA-8558	24	rs41646027	5.68E−06	*LPIN2*	0
		BOVINEHD2400010261	24	rs109172808	6.88E−06	*LPIN2*	0
		ARS-BFGL-NGS-83128	11	rs110743782	4.08E−05	*OTOF*	0
	Specific gravity	UA-IFASA-8558	24	rs41646027	2.62E−05	*LPIN2*	0
		BOVINEHD2400010261	24	rs109172808	2.81E−05	*LPIN2*	0
	Total solids Brix %	ARS-BFGL-NGS-83128	11	rs110743782	2.91E−06	*OTOF*	0
		ARS-BFGL-NGS-11057	11	rs109425927	7.78E−06	*OTOF*	0
		BOVINEHD2400010261	24	rs109172808	1.65E−05	*LPIN2*	0
		UA-IFASA-8558	24	rs41646027	1.70E−05	*LPIN2*	0
		BTB-02047078	6	rs43152213	3.30E−05	*ENSBTAG00000040324*	− 406,779
	Pneumonia	ARS-BFGL-NGS-48754	8	rs108973453	7.67E−06	*CAAP1*	0
		BOVINEHD0600010238	6	rs135767642	1.70E−05	*GPRIN3*	241,903
		HAPMAP31810-BTA-155140	2	rs42738873	4.46E−05	*ARHGAP15*	0
	Diarrhoea	BTA-41494-NO-RS	1	rs41641198	2.27E−05	*TBL1XR1*	320,947
		BTB-00647119	16	rs41812941	2.81E−05	*PRDM2*	320,495

*P* value = values are significant at the suggestive *P* value (*P* < 5 × 10^−5^), * = additionally significant at the Bonferroni genome wide significance *P* value threshold (i.e. Bonferroni *P* value threshold = 0.05/total no. of variants in analysis).

Sample sizes: Combined beef-suckler and dairy calves (Immunoglobulin G n = 1824, Pneumonia n = 1415 (77 case, 1338 control), Diarrhoea n = 1415 (237 case, 1178 control), Crude illness n = 1415 (357 case, 1058 control), Albumin n = 1838, Total protein n = 1838, Globulin n = 1833, Specific gravity n = 1839, Total solids Brix % n = 1836). Beef-suckler calves (Immunoglobulin G n = 679, Crude illness n = 686 (135 case, 551 control), Total protein n = 685, Globulin n = 681, Zinc sulphate turbidity n = 683, Specific gravity n = 686). Dairy calves (Immunoglobulin G n = 1143, Pneumonia n = 727 (33 case, 694 control), Diarrhoea n = 727 (176 case, 551 control), Albumin n = 1153, Total protein n = 1151, Globulin n = 1150, Specific gravity n = 1151, Total solids Brix % n = 1150).

**Figure 1:**
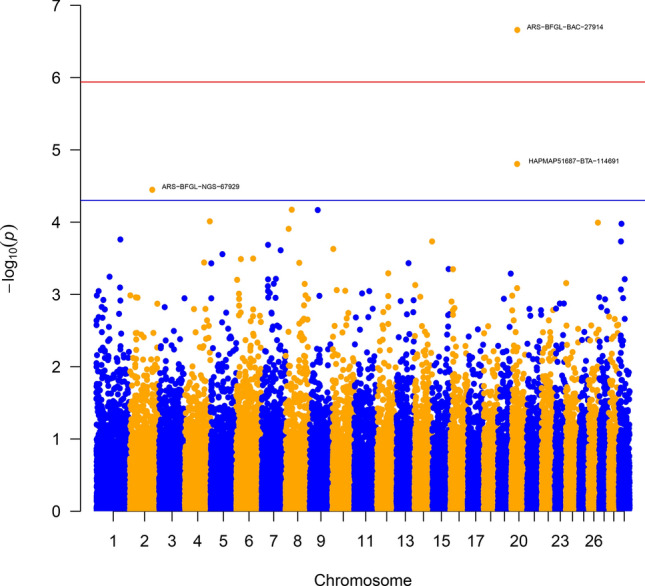
Manhattan plot for immunoglobulin G serum concentration in beef-suckler calves. The blue line indicates the suggestive *P* value threshold at *P* < 5 × 10^−5^. The red line indicates the Bonferroni genome wide significance *P* value threshold at *P* < 1.15 × 10^−6^.

In the analysis of beef-suckler calves, the heritability estimates of the passive immunity associated traits and the disease traits were low (range 0.02–0.10) (Table 2). There was one SNP which reached Bonferroni genome wide significance (ARS-BFGL-BAC-27914) (Table 3, Fig. 1) for an association with serum IgG concentration. This SNP was located within the intron of the *PARP8* gene, on chromosome 20 (Table 3, Supplementary Table S2). There were two SNPs in the serum IgG analysis, one SNP in the total protein analysis, four SNPs in the globulin analysis, four SNPs in the ZST analysis, one SNP in the specific gravity analysis and three SNPs in the crude illness analysis, which approached significance (*P* < 5 × 10^−5^) (Table 3).

In the analysis of dairy calves, the heritability estimates of the passive immunity associated traits and the disease traits were low to moderate (range 0.05–0.22) (Table 2). The trait with the highest heritability estimate was serum albumin concentration (0.22 ± 0.06) and the trait with the lowest heritability estimate was the serum total solids percentage measured by a Brix refractometer (0.05 ± 0.05) (Table 2). There were no SNPs which reached Bonferroni genome wide significance. However, there were three SNPs associated with serum IgG concentration, three SNPs associated with serum albumin concentration, four SNPs associated with serum total protein content, three SNPs associated with globulin concentration, two SNPs associated with specific gravity, five SNPs associated with total solids percentage from a Brix refractometer, three SNPs associated with incidents of pneumonia and two SNPs associated with diarrhoea occurrence, which were suggestively significant (*P* < 5 × 10^−5^) (Table 3).

## Discussion

To our knowledge, this is the first study to examine genetic associations with variables measuring the passive immune response and disease traits in beef-suckler and dairy calves in commercial herds. Failure of passive transfer of immunity in neonatal calves leads to greater incidents of disease, longer rearing periods, and increased use of antibiotic and anti-inflammatory treatments^45^. A meta-analysis and economic study has estimated the total cost of FPT in European beef and dairy production systems to be €80 and €60 per calf, respectively^1^. Therefore, reducing the prevalence of FPT in calves is warranted to improve animal welfare and augment the economic sustainability of beef and dairy farms. This is the first published study to examine genetic associations with variables measuring the passive immune response and disease traits in beef-suckler and dairy calves in commercial herds.

The GWAS analyses discovered several promising SNPs in all the passive immunity and disease trait analyses, and one SNP which reached genome wide significance in the serum IgG analysis in beef calves. This SNP is located in an intron of the *PARP8* gene, on chromosome 20. This gene is responsible for protein–protein interactions, protein-nucleic-acid interactions and the catalysation of the transfer of ADP-ribose from nicotinamide adenine dinucleotide onto target molecules, which consequently modifies the function of the target molecules^46,47^. It is of particular interest as it is a member of the *PARP* family and several *PARPs* are involved in the regulation of the adaptive immune system, inflammation, antiviral processes and activation of immune cells^46,47^. This gene, *PARP8*, is implicated in the breed specific development of immune competence in beef calves, as it displayed lower expression in the serum of Charolais-Limousin compared with Limousin-Friesian beef-suckler calves at 48 h post-birth^22^. Furthermore, increased expression of *PARP8* was observed in cultured bovine epithelial and stromal endometrial cells, following exposure to LPS for 6 hours^48^.

Several SNPs, including ARS-BFGL-BAC-27914, BTB-00212876, BTB-01120104, UA-IFASA-8558, ARS-BFGL-NGS-83128 SNP and ARS-BFGL-NGS-11057, were consistently found as suggestively associated with passive immunity using the several different tests, which increases their reliability as potential predictive markers for passively derived immunity. Interestingly, both the ARS-BFGL-NGS-83128 SNP and the ARS-BFGL-NGS-11057 SNP, which were suggestively associated with numerous indicators of passive immunity in dairy calves (total protein, globulin, total solids percentage from a Brix refractometer and total protein, total solids percentage from a Brix refractometer, respectively), are located within an intron of the *OTOF* gene, which is linked with a neurosensory non-syndromic recessive hearing loss^49^.

Other interesting SNPs include the ARS-BFGL-NGS-43453 SNP which reached a suggestive association with crude illness in the beef-suckler calves and is located in an intron of the *SMARCA2* gene on chromosome 8. This is noteworthy because *SMARCA2* is essential for the transcription of interferon-stimulated genes, which are important in the host response to viruses and intercellular pathogens^50^. Additionally, the BOVINEHD2400010261 SNP which tended to be associated with several passive immunity traits in both the combined beef and dairy calf population and the dairy calf population, is responsible for a missense mutation in the *LPIN2* gene, which is associated with a human autosomal recessive, auto-inflammatory disorder called Majeed syndrome^51^. Furthermore, the UA-IFASA-8558 SNP which was also suggestively associated with several passive immune traits in both the combined beef and dairy calf populations and the dairy calf population is likewise located in the *LPIN2* gene, but within an intron. The ARS-BFGL-NGS-50482 SNP which tended to be associated with pneumonia incidence in the combined beef-suckler and dairy calf population was closest to a gene, *CXCR4*, which was observed to have lower gene expression at 48 h post-birth in the serum of dairy calves (tube fed 5% of their body weight in colostrum, within one hour of birth) and Limousin-Friesian beef-suckler calves (that suckled their dams naturally, within one hour of birth) compared with 0 h (at birth)^22^. The BOVINEHD0600010238 SNP which was suggestively associated with pneumonia in the dairy calf population was closest to a gene, *GPRIN3*, which showed lower gene expression in the serum of dairy calves, Charolais-Limousin and Limousin-Friesian beef-suckler calves, at 48 h post-birth compared with at birth, and which showed higher expression in the dairy and the Limousin-Friesian beef-suckler calves serum at 168 h post-birth compared with 72 h post-birth^22^. The ARS-BFGL-NGS-11531 SNP which tended to be associated with albumin in both the combined beef and dairy calf and the dairy calf population, was located in an intron of the *DHRS12* gene. Interestingly, this gene may be involved in the development of the neonatal calves’ immune system as it has shown reduced expression in the serum of Charolais-Limousin beef-suckler calves at 48 h post-birth compared with at birth and it has breed specific expression levels as it was transcriptionally decreased in the serum of Charolais-Limousin compared with Limousin-Friesian beef-suckler calves at 48 h post-birth^22^ and displayed decreased expression in Jersey relative to Holstein–Friesian calves eight days following gradual weaning^52^. The SNP marker ARS-BFGL-NGS-67929, which was associated with IgG concentration in the serum of beef calves at a suggestive *P* value, was located within the *MREG* gene on chromosome 2. This gene, *MREG,* has been demonstrated to have higher expression in the serum of Charolais-Limousin and Limousin-Friesian beef-suckler calves at 48 h post-birth compared with at birth, and to have lower expression in the serum of Limousin-Friesian beef-suckler calves at 168 h post-birth compared with 72 h post birth^22^. Therefore, it appears to play a role in the acquisition of passively derived immunity in beef-suckler calves, and consequently, ARS-BFGL-NGS-67929 is a promising marker SNP for passive immune status. The HAPMAP54718-RS29022960 SNP which was approaching a significant association with serum IgG concentration in dairy calves, was located within an intron of the gene *ZNF292* which was observed to be transcriptionally decreased in the serum of Charolais-Limousin beef-suckler calves at 48 h post-birth compared with at birth^22^. Additionally, the BTB-00174357 SNP which tended to be associated with total serum protein in the combined beef and dairy population was closest to a gene *KIAA1324L* which showed reduced expression in the serum of Limousin-Friesian beef-suckler calves at 48 h post-birth compared with at birth^22^. Therefore, as the ARS-BFGL-BAC-27914, ARS-BFGL-NGS-50482, BOVINEHD0600010238, ARS-BFGL-NGS-11531, ARS-BFGL-NGS-67929, HAPMAP54718-RS29022960 and BTB-00174357 SNPs are either closest to, or within, a gene which has been observed to play a role in the development of immune competence in neonatal calves, these SNPs are promising candidates to confer superior immunity to calves.

A study on Canadian-Holstein cows has discovered 23 SNPs to be associated with serum IgG concentration^25^. One of these SNPs, BTA-03263-RS29011028, was found in the present study to be associated with serum globulin concentration of beef calves, at a suggestive *P* value (*P* < 5 × 10^−5^). Globulin concentration is as a proxy measure for IgG and can reflect the success of passive transfer in calves. This suggests a shared genetic background of immune-related traits across diverse cattle populations, and makes this variant, following validation, a promising candidate for inclusion as a genetic marker for IgG concentration in cattle. This SNP lies in an intergenic region on chromosome 21, with the closest gene being *PPP2R5C*, which is 20,692 nucleotides downstream of this variant. Apart from BTA-03263-RS29011028, there were no other SNPs significantly associated with IgG concentration in the Canadian study, which were also identified as associated with passive transfer or disease traits in the present study. The primary reason for the inconsistenices in the results between the two studies is likely due to the Canadian study examining the IgG natural antibodies in cows, whereas the present study focussed on passively derived IgG in calves. Other explanations include the effects of the different breeds used (commercial crossbred Irish beef and dairy breeds *versus* Canadian Holstein), the diverse locations and environmental conditions experienced by the animals, and the different SNP platforms used (IDB*v*3 SNP chip *versus* Illumina Bovine SNP50 BeadChip). Additionally, the different tests employed to determine IgG concentrations or passive immunity in serum can vary substantially in their accuracy, sensitivity and/or specificity (precision)^6,13^. Alternatively, it is plausible that some of SNPs in either the present study or the study on Canadian-Holstein cows^25^, are not truly related to passive immunity measurements and are simply correlated by chance.

A limitation to discovering reliable markers of disease resistance is the availability of accurate phenotype data^53^. Most health-related GWAS studies, including the disease traits in the present study, are heavily reliant on accurate disease reporting by producers; however, producers can often misdiagnose or fail to observe a disease case which leads to inaccurate phenotypic data. Schneider et al*.*^54^ reported that 60.6% of slaughtered feedlot cattle which never received treatment for BRD had lung lesions present and a study by Wittum et al*.*^55^ observed that 68% of slaughtered feedlot steers with no recorded history of BRD presented with lung lesions. Furthermore, health-related phenotypes are generally profoundly influenced by environmental and farm management factors^53^. In an attempt to control for these environmental and husbandry factors, only farms that had a minimum of 10 calves genotyped were utilised in this study and farm was included as a random factor in the phenotype models.

As immune responses and disease susceptibilities are complex traits which are lowly heritable, and possibly breed and pathogen specific^56^, they are likely governed by multiple genes. This means that large sample populations may be needed to discover reliable genetic markers, which if selected for, could possibly improve immunity and disease resistance. The relatively small sample size in this study is the probable reason for the lack of identification of a large quantity of SNPs which were significantly associated with passive immune status and disease traits. Additionally, the multitude of different breeds included in this study, particularly within the beef calf population, may have limited the ability to detect genetic associations with passively derived immunity and disease traits, despite the adjustment for breed structure which was performed in the phenotype models. This may account for the heritability estimates for the passive immune traits being substantially lower (range: 0.02–0.22) in this study relative to the heritability estimates for natural antibodies in the Holstein cows in the Canadian study (range: 0.27–0.31)^25^. This may also explain why many of the SNPs that were suggestively associated with either passive immunity or disease traits were not consistently found within the three populations; beef calves, dairy calves and the combined beef and dairy calf population. Furthermore, it is important to note that SNP-phenotype correlations do not guarantee causality, and consequently it is possible that some of the SNPs which are suggestively significant may be so by chance and may not be truly associated with the passive immunity or disease traits. Equally, SNPs which did not reach the suggestive *P* value association with the passive immune of disease traits may in fact be associated with those traits, if tested in a larger population.

In conclusion, several suggestive and significant SNP markers associated with passive immunity and disease resistance in Irish commercial beef-suckler and dairy calves, were discovered in this study. These SNPs could be tested in larger alternative beef and dairy populations and following validation, may contribute to Ireland’s national genomic selection breeding programme to select cattle with a greater resistance to disease.

## Supplementary information

Supplementary Table S1.

Supplementary Table S2.

## Data Availability

The datasets used and/or analysed during the current study are available from the corresponding author on reasonable request.
