# Supplementary material for: Genome wide association study of passive immunity and disease traits in beef-suckler and dairy calves on Irish farms
Source: Sci Rep. 2020 Nov 4;10:18998. doi: 10.1038/s41598-020-75870-4 (PMC7643155; doi:10.1038/s41598-020-75870-4)
Supplement: Supplementary file 2 — Supplementary Table S2. [file 41598_2020_75870_MOESM2_ESM.docx]

**Genome wide association study of passive immunity and disease traits in beef-suckler and dairy calves on Irish farms.**

Dayle Johnston^1^, Robert Mukiibi^2^, Sinéad M. Waters^1^, Mark McGee^1^, Carla Surlis^1^, Jennifer C. McClure^3^, Matthew C. McClure^3^, Cynthia G. Todd^1^ and Bernadette Earley^1^

^1^Animal & Grassland Research and Innovation Centre, Teagasc, Grange, Dunsany, Co. Meath, C15 PW93, Ireland.

^2^The Roslin Institute and Royal (Dick) School of Veterinary Studies, University of Edinburgh, Edinburgh, EH25 9RG, United Kingdom.

^3^Irish Cattle Breeding Federation, Cork, P72 X050, Ireland.

**Supplementary Table S2. Details of SNPs which reached either suggestive or genome wide significance in the GWAS studies examining passive immunity and disease traits in beef-suckler and dairy calves on Irish commercial farms.**

| SNP | Position | Chromosome | Location | RS No. | Associated gene | Gene name | Distance to gene |
| --- | --- | --- | --- | --- | --- | --- | --- |
| ARS-BFGL-BAC-27914 | 28364284 | 20 | intron | rs110897405 | *ENSBTAG00000004066* | *PARP8* | 0 |
| ARS-BFGL-NGS-100170 | 83739416 | 12 |  | rs109708871 | *ENSBTAG00000005925* | *SLC10A2* | -462315 |
| ARS-BFGL-NGS-110312 | 28184859 | 12 |  | rs110793235 | *ENSBTAG00000013984* | *KL* | -74444 |
| ARS-BFGL-NGS-11057 | 73014799 | 11 | intron | rs109425927 | *ENSBTAG00000009650* | *OTOF* | 0 |
| ARS-BFGL-NGS-114208 | 533815 | 1 |  | rs110082431 | *ENSBTAG00000045984* |  | 25324 |
| ARS-BFGL-NGS-114450 | 57619335 | 24 |  | rs109440690 | *ENSBTAG00000005738* | *ATP8B1* | -131562 |
| ARS-BFGL-NGS-114897 | 67750746 | 11 | downstream gene variant | rs110764285 | *ENSBTAG00000000141* | *NFU1* | 1970 |
| ARS-BFGL-NGS-11531 | 21315343 | 12 | intron | rs109028090 | *ENSBTAG00000016109* | *DHRS12* | 0 |
| ARS-BFGL-NGS-15820 | 21535801 | 11 | intron | rs110788172 | *ENSBTAG00000024044* | *CDKL4* | 0 |
| ARS-BFGL-NGS-4066 | 8695518 | 26 |  | rs109923400 | *ENSBTAG00000003529* |  | -5054 |
| ARS-BFGL-NGS-43453 | 42696770 | 8 | intron | rs110620477 | *ENSBTAG00000007494* | *SMARCA2* | 0 |
| ARS-BFGL-NGS-48754 | 17367679 | 8 | intron | rs108973453 | *ENSBTAG00000001223* | *CAAP1* | 0 |
| ARS-BFGL-NGS-50482 | 61134245 | 2 |  | rs110785912 | *ENSBTAG00000001060* | *CXCR4* | 447880 |
| ARS-BFGL-NGS-55396 | 76397552 | 10 | intron | rs110351463 | *ENSBTAG00000025450* | *SYNE2* | 0 |
| ARS-BFGL-NGS-57317 | 32823036 | 25 |  | rs110476838 | *ENSBTAG00000014417* |  | 334478 |
| ARS-BFGL-NGS-6195 | 13486389 | 18 | intron | rs109046420 | *ENSBTAG00000023745* | *BANP* | 0 |
| ARS-BFGL-NGS-67929 | 104555063 | 2 | intron | rs110780508 | *ENSBTAG00000013215* | *MREG* | 0 |
| ARS-BFGL-NGS-69831 | 41956061 | 7 | intron | rs42619441 | *ENSBTAG00000038284* |  | 0 |
| ARS-BFGL-NGS-83128 | 73068822 | 11 | intron | rs110743782 | *ENSBTAG00000009650* | *OTOF* | 0 |
| BOVINEHD0600010238 | 36747236 | 6 |  | rs135767642 | *ENSBTAG00000045966* | *GPRIN3* | 241903 |
| BOVINEHD0900029149 | 100554218 | 9 |  | rs109299906 | *ENSBTAG00000011593* | *QKI* | -73861 |
| BOVINEHD2400010261 | 37576220 | 24 | in gene, causes missense L [Leu] ⇒ P [Pro] | rs109172808 | *ENSBTAG00000012060* | *LPIN2* | 0 |
| BOVINEHD2900007001 | 24226648 | 29 |  | rs42465360 | *ENSBTAG00000014300* | *SLC6A5* | 338375 |
| BTA-03263-RS29011028 | 68356479 | 21 |  | rs29011028 | *ENSBTAG00000020192* | *PPP2R5C* | 20692 |
| BTA-41494-NO-RS | 90196132 | 1 |  | rs41641198 | *ENSBTAG00000021910* | *TBL1XR1* | 320947 |
| BTA-47238-NO-RS | 113215525 | 1 |  | rs110704582 | *ENSBTAG00000008307* | *PLCH1* | -144011 |
| BTB-00174357 | 33496136 | 4 |  | rs43383611 | *ENSBTAG00000004023* | *KIAA1324L* | 22817 |
| BTB-00212876 | 117412387 | 4 | intron | rs43420430 | *ENSBTAG00000021941* | *DPP6* | 0 |
| BTB-00647119 | 54775716 | 16 |  | rs41812941 | *ENSBTAG00000020552* | *PRDM2* | 320495 |
| BTB-01120104 | 41302649 | 4 |  | rs42277262 | *ENSBTAG00000002714* | *GNAI1* | -36497 |
| BTB-02047078 | 440021 | 6 |  | rs43152213 | *ENSBTAG00000040324* |  | -406779 |
| HAPMAP31810-BTA-155140 | 53330902 | 2 | intron | rs42738873 | *ENSBTAG00000032289* | *ARHGAP15* | 0 |
| HAPMAP39432-BTA-76145 | 48190681 | 6 |  | rs41596019 | *ENSBTAG00000019353* | *STIM2* | -184275 |
| HAPMAP40647-BTA-110965 | 13235237 | 4 | intron | rs41575187 | *ENSBTAG00000027134* | *DYNC1I1* | 0 |
| HAPMAP47694-BTA-67030 | 26358796 | 3 |  | rs43710738 | *ENSBTAG00000008022* | *PTGFRN* | 11015 |
| HAPMAP47742-BTA-80071 | 89384116 | 7 | intron | rs41656596 | *ENSBTAG00000009565* | *RASA1* | 0 |
| HAPMAP51687-BTA-114691 | 27659771 | 20 |  | rs41616927 | *ENSBTAG00000001241* | *ISL1* | 85644 |
| HAPMAP52014-BTA-90653 | 99569438 | 5 |  | rs41593661 | *ENSBTAG00000046268* |  | 30344 |
| HAPMAP54718-RS29022960 | 63574601 | 9 | intron | rs29022960 | *ENSBTAG00000046612* | *ZNF292* | 0 |
| UA-IFASA-8558 | 37587124 | 24 | intron | rs41646027 | *ENSBTAG00000012060* | *LPIN2* | 0 |
